# Systemic inflammation versus clinical demographics: patient age surpasses the platelet-to-lymphocyte ratio in differentiating secondary trigeminal neuralgia

**DOI:** 10.3389/fnagi.2025.1658854

**Published:** 2025-08-29

**Authors:** Zihao Zhang, Qingpei Hao, Tao Wang, Shijun Peng, Xin Chang, Yuepeng Wang, Jia Ouyang, Ruen Liu

**Affiliations:** ^1^Department of Neurosurgery, Peking University People’s Hospital, Beijing, China; ^2^Hebei Medical University, Shijiazhuang, Hebei, China; ^3^Department of Neurosurgery, The Second Affiliated Hospital, Hengyang Medical School, University of South China, Hengyang, Hunan, China

**Keywords:** trigeminal neuralgia, platelet-to-lymphocyte ratio, epidermoid cyst, age factors, meningioma

## Abstract

**Background:**

Objective biomarkers to differentiate trigeminal neuralgia (TN) subtypes are lacking. This study aimed to evaluate the utility of the platelet-to-lymphocyte ratio (PLR) and neutrophil-to-lymphocyte ratio (NLR) for distinguishing primary TN from secondary TN caused by meningiomas (STN-M) or epidermoid cysts (STN-E).

**Methods:**

In this retrospective study of 53 patients, analysis of covariance (ANCOVA) was used to compare adjusted biomarker levels while controlling for confounders. The diagnostic performance of these hematological markers and patient age was assessed using receiver operating characteristic (ROC) curve analysis, and their independent predictive values were determined by multivariable logistic regression to differentiate the two secondary TN types.

**Results:**

After adjusting for confounders, the mean PLR was significantly lower in the STN-M group compared to the STN-E group (*p* = 0.036), while NLR showed no significant difference. Notably, when comparing diagnostic performance for the secondary etiologies, patient age demonstrated a higher area under the curve (AUC = 0.962; 95% CI: 0.897–1.000) than PLR (AUC = 0.793; 95% CI: 0.614–0.972). Multivariable regression identified age as the most influential variable, showing a strong trend toward significance (*p* = 0.051), while PLR was not an independent predictor (*p* = 0.197).

**Conclusion:**

While this study identified PLR as a potential auxiliary biomarker, its most crucial finding is that the simple demographic feature of patient age is the primary and more powerful discriminator for differentiating STN-M from STN-E. This highlights that while novel biomarkers should be explored, the foundational importance of basic clinical parameters must not be overlooked in the pursuit of diagnostic precision.

## 1 Introduction

Trigeminal neuralgia (TN) is a debilitating neuropathic disorder defined by paroxysmal, electric shock-like facial pain. TN is broadly classified into primary TN (PTN), which includes classical TN caused by neurovascular compression (NVC) with morphological changes (e.g., nerve atrophy) and idiopathic TN with no compression or simple contact, and secondary trigeminal neuralgia (STN) resulting from underlying pathologies such as tumors or cysts (Cephalalgia, 2018; Ashina et al., 2019; Heinskou et al., 2019; Ujihara et al., 2020). While neuroinflammation is increasingly recognized as a key contributor to the pathophysiology across all TN types, marked by elevated pro-inflammatory cytokines that correlate with pain severity (Ostertag et al., 2023; Lai et al., 2025), the specific inflammatory signatures of different etiologies remain poorly understood.

Current TN subtyping relies heavily on magnetic resonance imaging (MRI) and clinical phenotyping, but those can be ambiguous, particularly when distinguishing between different causes of STN, especially between meningiomas and epidermoid cysts–two common causes of STN (Cruccu et al., 2016; Ujihara et al., 2020). Both pathologies trigger inflammatory responses, but through distinct mechanisms: meningiomas actively secrete pro-inflammatory cytokines (e.g., IL-6, TNF-α) that promote chronic perineural inflammation, whereas epidermoid cysts induce mechanical compression combined with intense focal inflammation when cyst contents (e.g., keratin) leak, activating immune cells and releasing localized cytokines (Haslund-Vinding et al., 2022; Zhang et al., 2022; Huang et al., 2024). This raises a question: can these distinct local inflammatory processes produce unique, measurable signatures in systemic circulation? Prior research on systemic inflammatory markers, such as the neutrophil-to-lymphocyte ratio (NLR) and platelet-to-lymphocyte ratio (PLR), has focused on immune dysregulation in conditions like diabetic neuropathy (Liu et al., 2017; Baka et al., 2021; Huang et al., 2024), leaving a gap regarding their utility in differentiating TN subtypes.

To address this gap, we hypothesized that the systemic inflammatory profiles reflected by NLR and PLR would differ significantly among PTN, meningioma-induced STN (STN-M), and epidermoid cyst-induced STN (STN-E). This study aimed to: (1) compare NLR and PLR levels across these three pathologically confirmed TN etiologies, and (2) evaluate the diagnostic accuracy of these hematological indices for discriminating TN subtypes. To our knowledge, this is the first study to investigate the potential of routine hematological markers to stratify TN etiologies, aiming to provide an objective, cost-effective, and universally available tool to enhance preoperative diagnostic precision.

## 2 Materials and methods

### 2.1 Patient selection

Using International Classification of Diseases, Tenth Revision (ICD-10) codes (e.g., G50.0), we identified a cohort of consecutive patients who underwent surgery for TN at Peking University People’s Hospital between January 2018 and December 2023. Following a retrospective review of their medical records, a total of 53 patients who met all inclusion and exclusion criteria were included in the final analysis.

Patients were excluded based on the following criteria: (1) STN attributed to causes other than meningioma or epidermoid cyst, such as multiple sclerosis, other types of intracranial tumors, or post-herpetic neuralgia; (2) any evidence of active systemic or localized infection, or diagnosed systemic inflammatory or autoimmune diseases at the time of blood sampling; (3) use of corticosteroids, immunosuppressants, or other immunomodulatory drugs within the 3 months prior to admission; (4) history of other malignancies or hematological disorders; (5) severe, uncontrolled comorbidities (e.g., cardiac, pulmonary, or renal failure); (6) incomplete clinical and laboratory data.

The study was approved by the Ethics Committee of Peking University People’s Hospital (Approval Number: 2022PHB099-002), which waived the requirement for individual informed consent due to the retrospective nature of the study and the use of de-identified patient data. All procedures were performed in accordance with the Declaration of Helsinki.

### 2.2 Surgical procedure and pathological confirmation

All surgical interventions were performed by a consistent senior neurosurgical team. Depending on the preoperative diagnosis, patients underwent either MVD for PTN or tumor resection for STN. The ultimate goal of surgery in the context of this study was to obtain a definitive etiological diagnosis. All cases of STN were confirmed via postoperative histopathology, while PTN cases were confirmed by intraoperative identification of NVC in the absence of other pathologies.

### 2.3 Data collection and study variables

For each patient, we retrospectively collected demographic data (age at surgery, sex) and key clinical characteristics from their medical records. Disease duration was defined as the time in years from the self-reported onset of symptoms to the date of surgical intervention. Pain intensity upon admission was assessed using the Visual Analog Scale (VAS). Baseline hematological parameters were obtained from the first complete blood count performed within 24 h of hospital admission, prior to any surgical procedures or corticosteroid administration. We extracted the absolute counts of neutrophils, lymphocytes, and platelets (all in ×10^9^/L). From these values, we calculated two key inflammatory indices: the NLR, defined as the absolute neutrophil count divided by the absolute lymphocyte count, and the PLR, defined as the absolute platelet count divided by the absolute lymphocyte count.

Patients were stratified into three distinct etiological groups based on definitive diagnostic findings. The STN groups–STN-M and STN-E–were confirmed by postoperative histopathological analysis of resected tissue. The PTN group was confirmed by intraoperative exploration that revealed NVC without any other identifiable pathology.

### 2.4 Statistical analysis

All statistical analyses were performed using SPSS version 27 (IBM Corp., Armonk, NY), with data presented as mean ± standard deviation (SD) for continuous variables and as counts and percentages for categorical variables. A two-tailed *p*-value of less than 0.05 was considered statistically significant. Prior to analysis, assumptions for parametric testing were verified; the normality of data distribution was assessed using the Shapiro-Wilk test, and the homogeneity of variances was confirmed with Levene’s test. Baseline demographic and clinical characteristics were compared across the three etiological groups using one-way analysis of variance (ANOVA) or the chi-square test (or Fisher’s exact test, as appropriate).

To compare the primary inflammatory markers (PLR and NLR) while adjusting for confounding effects, analysis of covariance (ANCOVA) was employed. Covariates for this model–age and disease duration–were selected based on their established clinical relevance as potential confounders, and significant differences across the study groups were also noted. If the overall ANCOVA result was significant, *post hoc* pairwise comparisons were planned using the Bonferroni correction.

Receiver operating characteristic curve analysis was employed to evaluate the discriminative ability of selected parameters in distinguishing between meningioma and epidermoid cyst. The optimal cut-off value for each predictor was determined using the Youden’s index. Finally, a binary logistic regression model was constructed to assess the independent predictive value of age and PLR. Multicollinearity between predictors was assessed using the variance inflation factor (VIF) to ensure model stability.

## 3 Results

### 3.1 Patient baseline characteristics

A total of 53 patients were included in this retrospective study, comprising 24 with PTN, 16 with STN-E, and 13 with STN-M. No significant differences were observed among the three groups regarding sex, pain laterality, the number of affected trigeminal nerve divisions, or baseline VAS scores (all *p* > 0.05). However, the groups differed significantly in age (*p* < 0.001), disease duration (*p* = 0.046), and hospitalization duration (*p* < 0.001).

A review of the group means in Table 1 indicates that patients in the STN-M group were the oldest and had the longest hospitalization stays, whereas patients with PTN reported the longest disease duration. Baseline platelet counts did not differ significantly among the groups (*p* = 0.054), though the highest mean level was observed in the STN-E group. The detailed baseline characteristics of the study population are presented in Table 1.

**TABLE 1:** Baseline characteristics of the study population.

Characteristic	PTN	STN-E	STN-M	*P*-value
Number	24	16	13	
Sex, no. (%)				0.746
Male	7 (29.2%)	6 (37.5%)	3 (23.1%)	
Female	17 (70.8%)	10 (62.5%)	10 (76.9%)	
Age (y)	58.50 ± 11.22	43.06 ± 10.17	63.46 ± 7.83	**<0.001**
Disease duration (y)	8.53 ± 10.02	3.91 ± 3.26	3.07 ± 2.36	0.046
VAS score	8.83 ± 0.87	8.38 ± 0.89	8.46 ± 0.88	0.224
Hospitalization duration (d)	7.92 ± 2.17	8.19 ± 2.04	12.92 ± 5.50	**<0.001**
Laterality				0.172
Left	4 (16.7%)	7 (43.8%)	4 (30.8%)	
Right	20 (83.3%)	9 (56.3%)	9 (69.2%)	
Affected division, no. (%)				0.219
V1, V2	3 (12.5%)	1 (6.3%)	0 (0.0%)	
V1, V2, V3	0 (0.0%)	2 (12.5%)	1 (7.7%)	
V2	10 (41.7%)	9 (56.3%)	8 (61.5%)	
V3	6 (25.0%)	1 (6.3%)	0 (0.0%)	
V2, V3	5 (20.8%)	3 (18.8%)	4 (30.8%)	
Number of affected branches, no. (%)				0.551
1	16 (66.7%)	10 (62.5%)	8 (61.5%)	
2	8 (33.3%)	4 (25.0%)	4 (30.8%)	
3	0 (0.0%)	2 (12.5%)	1 (7.7%)	
Acute exacerbation, no. (%)				0.276
Without	5 (20.8%)	5 (31.3%)	6 (46.2%)	
With	19 (79.2%)	11 (68.8%)	7 (53.8%)	
Neutrophil count (10^9^/L)	3.50 ± 1.09	3.48 ± 1.03	3.54 ± 1.13	0.988
Lymphocyte count (10^9^/L)	1.61 ± 0.46	1.61 ± 0.53	1.64 ± 0.54	0.446
Platelet count (10^9^/L)	207.33 ± 60.10	236.00 ± 59.59	179.54 ± 64.13	0.054

Data are presented as mean ± standard deviation for continuous variables and as number (%) for categorical variables. Comparisons among the three groups–primary trigeminal neuralgia (PTN), secondary trigeminal neuralgia due to epidermoid cyst (STN-E), and secondary trigeminal neuralgia due to meningioma (STN-M)–were performed using one-way ANOVA for continuous variables and the Fisher’s exact test for categorical variables. Statistically significant *p*-values (*p* < 0.05) are highlighted in bold. VAS, visual analog scale.

### 3.2 Adjusted comparison of inflammatory biomarkers

**FIGURE 1:**
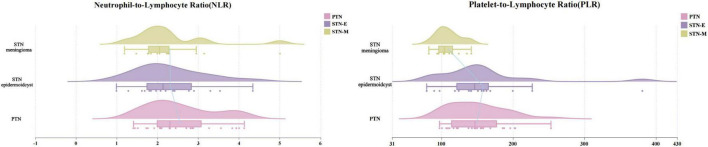
Distribution of inflammatory biomarkers across trigeminal neuralgia subtypes. This figure displays raincloud plots comparing the distribution of the Neutrophil-to-Lymphocyte Ratio (NLR) and Platelet-to-Lymphocyte Ratio (PLR) among the three study groups. Each plot combines three visual elements: (1) a kernel density plot (the “cloud”) on top, showing the probability distribution of the data; (2) a standard box plot in the middle, indicating the median, interquartile range, and outliers; and (3) a swarm plot at the bottom (the “rain”), showing the individual data points for each patient without overlap. PTN, primary trigeminal neuralgia; STN-E, secondary trigeminal neuralgia caused by epidermoid cyst; STN-M, secondary trigeminal neuralgia caused by meningioma.

After adjusting for age and disease duration, ANCOVA revealed a significant difference in the adjusted mean PLR among the three etiological groups (F(2, 48) = 3.787, *p* = 0.030) (Table 2). *Post hoc* analysis using Bonferroni correction indicated that this difference was driven by a significantly lower adjusted mean PLR in the STN-M group compared to the STN-E group (*p* = 0.036). No other significant pairwise differences for PLR were observed. In contrast, after the same adjustments, there was no significant difference in the adjusted mean NLR among the groups (F(2, 48) = 0.692, *p* = 0.506) (Figure 1).

**TABLE 2:** Adjusted comparison of inflammatory biomarkers across TN subtypes.

Biomarker	PTN (*n* = 24)	STN-E (*n* = 16)	STN-M (*n* = 13)	F-statistic	*P*-value
PLR	146.77 (125.90–167.63)	165.74^a^	105.72^b^	3.787	0.03
NLR	2.60 (2.21–2.98)	2.25 (1.71–2.80)	2.31 (1.74–2.87)	0.692	0.506

Data are presented as adjusted mean (95% CI) derived from one-way analysis of covariance (ANCOVA), controlling for age and disease duration. Different superscript letters (^a^, ^b^) in the same row indicate a statistically significant difference (*p* < 0.05) between groups based on *post hoc* analysis with Bonferroni correction. PTN, primary trigeminal neuralgia; STN-E, secondary trigeminal neuralgia due to epidermoid cyst; STN-M, secondary trigeminal neuralgia due to meningioma; PLR, platelet-to-lymphocyte ratio; NLR, neutrophil-to-lymphocyte ratio; CI, confidence interval.

### 3.3 Diagnostic performance of age and PLR for differentiating STN

To differentiate between patients with STN-M and STN-E, ROC curve analysis was performed. Age demonstrated a high level of discriminatory power, with an area under the curve (AUC) of 0.962 (95% CI: 0.897–1.000; *p* < 0.001). Based on the Youden’s index, the optimal cut-off value for age was 47.5 years, which yielded a sensitivity of 100.0% and a specificity of 84.6%.

**FIGURE 2:**
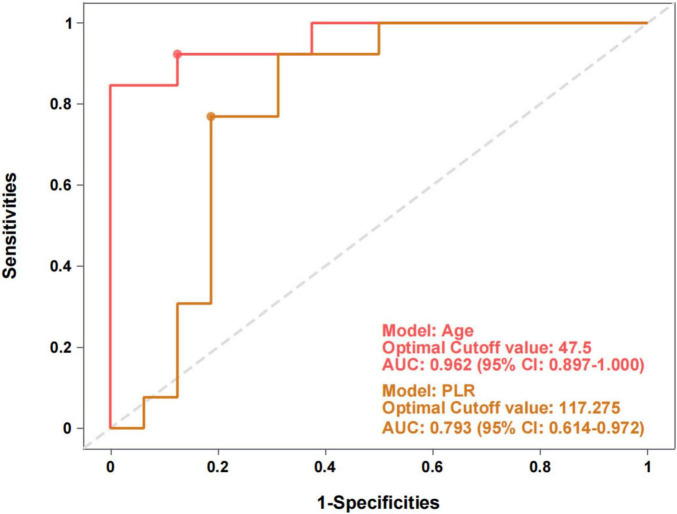
Diagnostic performance of age and PLR for differentiating STN etiologies. This figure shows the Receiver Operating Characteristic (ROC) curves comparing the ability of patient age and the Platelet-to-Lymphocyte Ratio (PLR) to differentiate between meningioma- and epidermoid cyst-induced STN. The Area Under the Curve (AUC) represents the overall diagnostic accuracy of each model, with a value closer to 1.0 indicating higher accuracy. Optimal cutoff values for each predictor were determined to maximize sensitivity and specificity. STN, secondary trigeminal neuralgia; AUC, area under the curve; CI, confidence interval.

Platelet-to-lymphocyte ratio also showed diagnostic utility, with an AUC of 0.793 (95% CI: 0.614–0.972; *p* = 0.007). The optimal PLR cut-off was 117.28, corresponding to a sensitivity of 68.8% and a specificity of 92.3%. The ROC curves for both age (after reverse scoring) and PLR are displayed in Figure 2.

### 3.4 Independent predictors of etiology in STN

A binary logistic regression model was constructed to assess independent predictors for differentiating STN-M from STN-E. Multicollinearity diagnostics were examined prior to the analysis; the VIF for both age (VIF = 1.13) and PLR (VIF = 1.21) were substantially below the commonly used cutoff of 5, indicating a low risk of multicollinearity. The overall model was statistically significant (Omnibus test, *p* < 0.001) and explained a substantial portion of the variance in etiology (Nagelkerke R^2^ = 0.868). In the multivariable analysis, age showed a statistical trend toward significance (adjusted odds ratio [aOR] = 1.56; 95% CI: 1.00–2.43; *p* = 0.051), indicating that older age may be associated with an increased likelihood of meningioma relative to epidermoid cyst, though this did not reach conventional statistical significance. In contrast, after adjusting for age, PLR was not a significant independent predictor (aOR = 0.95; 95% CI: 0.88–1.03; *p* = 0.197). Overall, the model correctly classified 89.7% of cases, with a sensitivity of 87.5% for identifying epidermoid cysts and a specificity of 92.3%.

## 4 Discussion

To our knowledge, this is the first study to compare systemic inflammatory markers, NLR and PLR, across distinct etiologies, including primary, meningioma-associated, and epidermoid cyst-associated forms. Our primary finding is twofold. First, after adjusting for age and disease duration, PLR was significantly lower in patients with STN-M than in those with STN-E, whereas NLR showed no intergroup differences. Second, and more importantly, simple patient age emerged as a more powerful discriminator for these two secondary etiologies than the biological marker. ROC analysis confirmed that age (AUC = 0.962) offered higher diagnostic performance compared to PLR (AUC = 0.793), establishing it as the principal predictor.

A particularly intriguing finding of our study was the significantly lower PLR in patients with STN-M compared to those with STN-E. This result is compelling, as one might intuitively hypothesize that a neoplastic process would elicit a stronger systemic inflammatory response. This observation leads to several potential pathophysiological mechanisms. For meningiomas, one plausible mechanism is the local consumption of platelets within the tumor microenvironment. Meningiomas are well-documented to express and secrete high levels of vascular endothelial growth factor (VEGF), a potent pro-angiogenic mediator (Ding et al., 2008; Schmid et al., 2010). Given that VEGF directly induces platelet activation and aggregation, it is conceivable that tumor-derived VEGF in meningiomas leads to localized platelet sequestration and consumption at the tumor site, thereby leading to a relative decrease in circulating platelets which lowers systemic PLR (Liao et al., 2023; Huang et al., 2024; Le Chapelain et al., 2024).

In stark contrast, the inflammatory stimulus from an epidermoid cyst may drive a systemic increase in platelets. The keratin debris within these cysts acts as a potent foreign body, capable of triggering a chronic inflammatory cascade that systemically stimulates thrombopoiesis. This process is likely mediated via an interleukin-6 (IL-6) dominant signaling cascade, which is known to increase hepatic thrombopoietin production and subsequent platelet generation (Liao et al., 2023; Köse et al., 2025; Lai et al., 2025). Therefore, the opposing effects on PLR likely reflect two fundamentally different host-tumor interactions: localized, VEGF-mediated platelet consumption in meningiomas versus systemic, cytokine-driven platelet overproduction triggered by epidermoid cysts.

The lack of a significant difference in NLR among the TN subtypes is also a noteworthy finding, particularly as NLR is often considered a reliable marker in other neuropathic pain conditions, such as diabetic neuropathy. This discrepancy may be attributable to the underlying pathology of TN, especially in cases secondary to benign tumors and cysts. The core mechanism in these conditions is often a combination of chronic mechanical compression and localized perineural inflammation, rather than a robust, systemic inflammatory state characterized by massive neutrophil mobilization. Unlike severe systemic infections or metabolic disorders where a systemic neutrophilic response is prominent, the inflammatory signature in these TN subtypes may be too localized to significantly alter the systemic neutrophil-to-lymphocyte ratio. This is consistent with evidence that NLR is more responsive to acute systemic inflammatory states than to chronic or localized processes (Buonacera et al., 2022; Li and Shao, 2025). Therefore, it is plausible that NLR, a marker more reflective of acute systemic inflammation, lacks the sensitivity to differentiate between TN etiologies that are primarily defined by localized structural and inflammatory changes.

A pivotal finding of this study was the strong diagnostic performance of patient age in distinguishing between secondary etiologies, with its AUC of 0.962 surpassing that of PLR (AUC = 0.793). This result aligns with the established epidemiological profiles of these pathologies: epidermoid cysts are congenital lesions that typically manifesting in younger adults, whereas meningiomas are most prevalent in middle-aged and older populations–a demographic distinction clearly reflected in our cohort. The influential role of age was further confirmed by the multivariable logistic regression analysis. When both age and PLR were included in the model, PLR lost its independent predictive significance (*p* = 0.197). In contrast, age remained the most influential variable, showing a strong trend toward statistical significance (*p* = 0.051). This suggests that the diagnostic utility observed for PLR in the univariable analysis is largely confounded by its intrinsic association with the age-dependent nature of the underlying pathologies.

Within the context of our cohort, our findings offer a pragmatic heuristic for the preoperative differential diagnosis of STN. Patient age emerged as the most powerful, zero-cost initial screening tool. Our data indicate that an age greater than 47.5 years is a strong predictor for a meningioma over an epidermoid cyst, a finding that can immediately guide clinical suspicion. PLR, in turn, serves as an auxiliary biomarker, particularly in diagnostically ambiguous scenarios. For instance, in an older patient whose imaging findings are atypical and could suggest an epidermoid cyst, a PLR value below the 117.28 cut-off provides additional evidence for a meningioma diagnosis, owing to its high specificity of 92.3%. It is crucial to emphasize that these parameters are not intended to replace MRI as the gold standard for diagnosis. Rather, they should be regarded as inexpensive and widely available auxiliary tools that can enhance diagnostic confidence, particularly in cases with atypical imaging findings, or assist in preoperative planning.

This study possesses several strengths, including its novel design as the first to systematically compare NLR and PLR across pathologically confirmed TN subtypes. The accuracy of our group categorization was ensured by postoperative pathological verification, and the robustness of our statistical approach was enhanced by adjusting for key confounders. Furthermore, all procedures were performed by a consistent, senior surgical team, minimizing operator-related bias. Nevertheless, we must acknowledge several limitations. First and foremost, the relatively small sample size (*n* = 53), particularly within the STN-M subgroup (*n* = 13), limits statistical power and the generalizability of our findings. And this study also lacks external validation or internal cross-validation procedures to confirm the robustness of the regression model. Given the relatively small sample size, especially within the STN subgroups, the reported classification accuracy of 89.7% may be inflated due to overfitting. Future studies should incorporate k-fold cross-validation or independent test sets to ensure the generalizability and stability of the model across diverse patient populations. Second, the retrospective, single-center design introduces inherent risks of selection bias. Furthermore, a key limitation of this study is that it included only surgically treated patients, which may also introduce selection bias and limit the generalizability of our findings. As such, the results primarily apply to patients with surgically confirmed TN subtypes. Future studies should aim to validate these findings in broader TN populations, including non-surgical cases, to assess whether the observed hematological trends are consistent across treatment modalities. Finally, this study relied on peripheral hematological markers as proxies for the local inflammatory state. While these markers are highly accessible, they can be influenced by unmeasured subclinical confounders, and they preclude the establishment of a direct causal link between the specific pathologies and the observed systemic inflammatory profiles without analysis of the local milieu.

Building upon these findings and limitations, future research should prioritize the validation of our results through large-scale, multi-center prospective studies to confirm the diagnostic thresholds for age and PLR. Furthermore, mechanistic studies are warranted to elucidate the precise link between systemic inflammatory markers and the local pathological microenvironment. Such investigations should ideally involve the simultaneous analysis of peripheral blood markers with local inflammatory mediators in CSF or resected tissue, focusing on cytokines previously implicated in TN pathophysiology, such as IL-6 and TNF-α (Ericson et al., 2019; Lai et al., 2025; Xu et al., 2025), to bridge the gap between systemic and local inflammation.

## 5 Conclusion

This study demonstrates that while the PLR shows potential for differentiating STN-M versus STN-E, its diagnostic value is substantially surpassed by the simple demographic feature of patient age. These findings underscore the potential of leveraging inexpensive, widely available clinical and hematological parameters as auxiliary tools to enhance diagnostic precision. However, they also affirm that a patient’s classic demographic profile and standard neuroimaging remain the cornerstone of clinical decision-making. Future research is required to elucidate the complex pathophysiological mechanisms that underlie these biomarker associations and to validate these findings in larger, prospective cohorts.

## Data Availability

The data analyzed in this study is subject to the following licenses/restrictions: The datasets generated and/or analyzed during the current study are available from the corresponding author upon reasonable request. Requests to access these datasets should be directed to liuruen@pku.edu.cn.
